# Barriers to optimal chronic pain management in refugees: a scoping review

**DOI:** 10.1186/s12889-026-26206-w

**Published:** 2026-01-13

**Authors:** Emma Victoria Marianne Bilney, Maris Vasenius, John Mitchell, Julia Downing, Pierre-Michel Francois, Stephen McCall, Patrice Forget

**Affiliations:** 1 School of Medicine, Medical Sciences and Nutrition, University of Aberdeen, Aberdeen, UK; 2 University of Helsinki, Helsinki, Finland; 3 Anesthesiology, CHU (Centre Hospitalier Universitaire) UCLouvain (Université Catholique deLouvain) Namur - Godinne, Godinne, Belgium; 4 International Children’s Palliative Care Network, Lewins Mead, Bristol, UK; 5 Queen Astrid Military Hospital, Brussels, Belgium; 6 Center for Research On Population and Health, Faculty of Health Sciences, American University of Beirut, Beirut, Lebanon; 7 Aberdeen Centre for Women’s Health Research, Institute of Applied Health Sciences, School of Medicine, Medical Sciences and Nutrition, University of Aberdeen, Aberdeen, UK; 8 Aberdeen Centre of Musculoskeletal Health (Epidemiology Group), Institute of Applied Health Sciences, School of Medicine, Medical Sciences and Nutrition, University of Aberdeen, Aberdeen, UK; 9 Department of Anaesthesia, NHS Grampian, Aberdeen, UK; 10 Pain and Opioids After Surgery (PANDOS) Research Groups, European Society of Anaesthesiology and Intensive Care, Brussels, Belgium; 11 IMAGINE UR UM 103, Montpellier University, Anesthesia Critical Care, Emergency and Pain Medicine Division, Nîmes University Hospital, 30900 Nîmes, France

**Keywords:** Chronic pain, Refugees, Management

## Abstract

**Background:**

In 2023, there were 36.4 million refugees worldwide, many of whom experience physical injuries, human rights abuses and psychological distress during displacement. However, their healthcare needs during and after resettlement are rarely met. Among them, chronic pain and its effective management, are under-recognized but prevalent problems of humanitarian care. The aim of this scoping review was to improve our understanding of the challenges in treating and managing chronic pain in refugee populations. Specifically, this scoping review aims to address the following objectives: (i) to identify and systematically map available evidence in the literature on chronic pain treatment for refugees (ii) to identify barriers to optimal pain management for refugees, and (iii) identify gaps within the knowledge base.

**Methods:**

Systematic searches were conducted in five online databases to identify studies that examined or described treatment of chronic pain in refugees. Narrative analysis was conducted to describe the relevant themes extracted from the included literature.

**Results:**

28 English-language articles were included, from which two general themes were identified regarding chronic pain treatment in refugees: (i) Pain Severity, and (ii) Psychological Correlates of Pain. Three themes were identified relating to barriers to optimal chronic pain management for refugees: (i) Sociocultural Considerations, (ii) Access to Adequate Healthcare and Medication, and (iii) Integrative Pain Management Interventions. Literature and practice gaps in the literature were also elaborated.

**Conclusion:**

It is essential to address barriers to optimal chronic pain management through multidisciplinary approaches that prioritize cultural competence and are tailored to the unique pre- and post-migration experiences of refugees. Furthermore, high-quality controlled studies are needed to investigate the effectiveness of context-specific multidisciplinary pain management interventions.

**Supplementary Information:**

The online version contains supplementary material available at 10.1186/s12889-026-26206-w.

## Introduction

According to the UN Refugee Agency (UNHCR), in 2023, there were 36.4 million refugees globally [1, 2]. The displacement of refugees often involves a rushed escape from their home, leaving most possessions behind and experiencing human rights abuses, physical injuries sustained during escape, and witnessing the loss or assault of family and friends. Whilst disabilities are often caused by such experiences in their home country, particularly for women, migration and resettlement in a host country present a further challenge. Many refugees spend months or years in conditions that fall short of basic humanitarian standards [3]. Whilst legal and socio-economic situations vary, many refugees are at a high risk of inadequate healthcare and are disproportionately affected by ill-health and disease due to their transitory status and lack of access to basic needs [4, 5]. Chronic pain, a complex and enduring human condition that persists beyond the typical healing trajectory of tissue injuries, is a commonly noted health issue among refugees who have often experienced trauma [6, 7].

As defined by the International Association for the Study of Pain (IASP), chronic pain is persistent or recurrent pain that lasts longer than three months [8]. Research has found a high prevalence of chronic pain in refugee populations, influenced by pre-migration factors and post-migration challenges [9]. The treatment of chronic pain is complex in general, as it should involve a combination of various therapeutic approaches (including pharmacological interventions, psychological interventions and integrative strategies) [10]. Multidisciplinary interventions can reduce pain intensity, improve functional impairment, and alleviate psychosocial symptoms in chronic pain patients from refugee or immigrant backgrounds [11]. Too often, these treatments are not available and refugees receive neither the necessary healthcare required for functionality nor the recommended integrative services to manage chronic pain [11]. This underscores their unmet healthcare needs during resettlement. Thus, increasing clinical awareness of chronic pain and its management is crucial to aiding refugee health services.

Additionally, refugee populations are diverse, often suffering from multiple post-migration living difficulties such as language differences, family concerns, loneliness, discrimination, legal status, duration of residence, and financial hardship, regularly combined with posttraumatic stress symptoms, depression or anxiety [12]. Migration and displacement induced multicultural heterogeneity poses amplified challenges for patients seeking to access existing healthcare services for chronic pain treatment and can lead to added complexities in the treatment itself.

The purpose of this scoping review was to understand the challenges of treatment of chronic pain in refugee populations by addressing the following objectives: (i) to map the existing literature on chronic pain treatment for refugees, (ii) to identify challenges in the treatment of chronic pain in refugees, and (iii) to identify gaps within the knowledge base.

## Methods

### Search strategy, study selection, and exclusion criteria

A scoping review was conducted to identify relevant academic literature focusing on chronic pain treatment in refugee populations. This review was informed by Arksey and O’Malley’s [13] methodological framework and recommendations from Levac et al. [14] and Peters et al. [15]. Reporting was informed by PRISMA guidelines for scoping reviews [16] (detailed checklist in Additional File 1).

A search strategy was implemented to find relevant publications, which were screened by two authors. The first title and abstract screen was conducted by MV in January 2021 and then a second title and abstract screen was conducted by EB, which updated the results in October 2024. Studies were selected using our predetermined inclusion and exclusion criteria. Papers were included if (1) study subjects were refugees, (2) the article investigated chronic pain, (3) and the article included management or treatment of chronic pain. Publications were excluded if: (1) the reviewers were unable to obtain an English translation of the article, (2) the full-text of the article was unavailable, (3) the article only addressed psychological interventions or treatments (4) the article only addressed torture survivors decided by authors because of the complex and pain sequelae experienced by survivors of torture that may not be applicable to the general refugee population (5) the article was a study protocol. No restrictions were added to the location or year of publication. Additionally, all study designs and articles were eligible for inclusion due to the limited number of empirical research studies on this topic. Subsequently, authors EB and MV independently extracted the data from the included publications, initially by MV in January 2021 and then by EB in October 2024. Subsequently, the authors organized, summarized, and presented the results.

The search parameters included studies from peer-reviewed literature relevant to the management or treatment of chronic pain in refugees. To locate peer-reviewed literature the following five electronic bibliographic databases were searched: PubMed/MEDLINE, Web of Science, Cochrane Database of Systematic Reviews, Scopus, and APA PsycInfo, in January 2021 and updated in October 2024.

An example of our search term strategy can be found in Additional File 2. Broad search terms, with variations and relevant synonyms, were deliberately chosen to capture the scope of the literature. The search term strategy was systematically used across all queried databases.

### Data extraction

Data were extracted independently by authors MV and EB (conducting a last update in October 2024), summarized in a Microsoft Excel spreadsheet. Data extraction of study characteristics included descriptive characteristics (authors, year, title, study design, location, key aims and objectives, main study findings), and participant characteristics (sample size, gender, nationality). In addition, content was extracted on the topic of interest for this review, comprising the current state of the literature in chronic pain treatment for refugees and challenges to optimal chronic pain management.

### Data synthesis and integration

Evidence was synthesized narratively by authors EB and MV. Reflective discussion and iterative review of the themes occurred at update with authors EB and PF. Content for challenges in chronic pain management was grouped into meaningful themes and sub-themes. Furthermore, content on source article objectives, findings, and limitations were grouped into themes which do not directly focus on challenges but on chronic pain treatment in refugee populations in general.

## Results

### Study characteristics

**Fig. 1:**
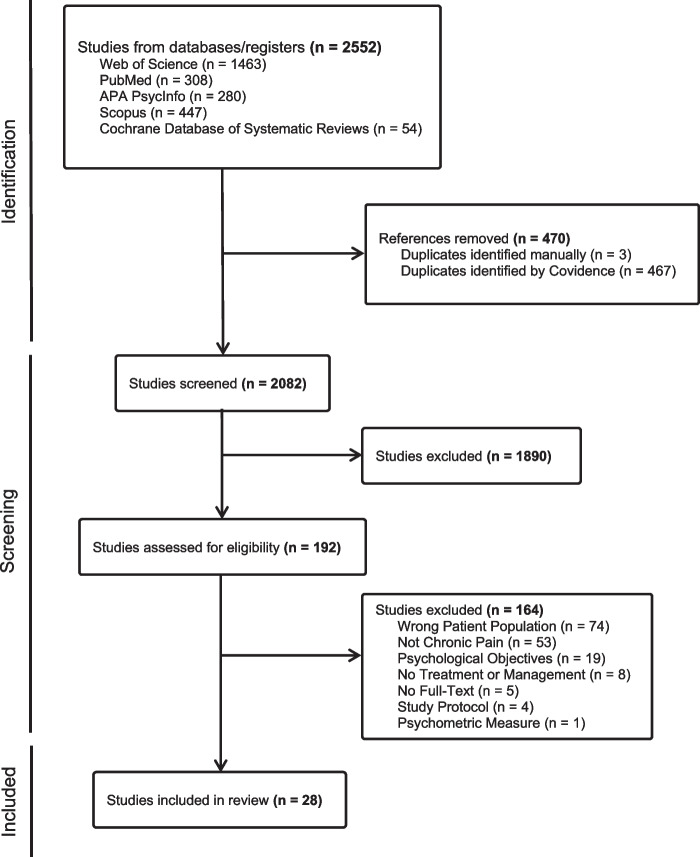
PRISMA Study Selection Flow Diagram

The database searches yielded 2,552 potential articles. Figure 1 illustrates the study selection process using a PRISMA diagram. After exclusion of duplicates, and application of inclusion and exclusion criteria, 28 articles were selected for inclusion, all of which were academic peer-reviewed publications. Table 1 details the included study designs, participant characteristics, and relevant outcomes. Of the included sources, twelve were quantitative studies, seven were qualitative studies, one was a mixed methods design, and eight were review articles. The quantitative and qualitative parts of the mixed methods study were separated for analysis and included in the relevant sections.

**Table 1:** Summary of Relevant Findings in the Included Literature

Author	Study Location	Participant Nationalities	Refugee Sample Size	Key aims and objectives	Methods	Summary of relevant findings	Themes
Altun et al [9].	Australia	North African, Middle Eastern, South-East Asian, North-East Asian, Southern and Central Asian, Americas, Sub-Saharan African	310	To examine pre- and post-migration factors and their association with chronic pain among resettled humanitarian refugee women in Australia	Quantitative- Secondary Survey and Interview Data from a Longitudinal Cohort Study	This study reported a high prevalence of chronic pain during the initial years of resettlement. Chronic pain was reported in 45% of women participating in the study. Pre-migration factors, such as age and migration pathway, were identified as predictors of greater self-reported chronic pain. Post-migration factors, such as rurality of settlement, poorer general health, and perceived discriminatory experiences, were identified as predictors of greater self-reported chronic pain	Pain Severity; Sociocultural Considerations; Access to Adequate Healthcare and Medication
Negron [17]	United Kingdom	Sri Lankan, Iranian, Eritrean, Iraqi, Congolese (Democratic Republic of Congo), Cameroonian**	173*	The discuss and evaluate refugees’ experiences with the Wellbeing and Self-Care project for refugees and asylum seekers who suffer from persistent pain related to their traumatic experiences	Quantitative – Case Study	Most participants reported at least one area of physical pain and widespread pain was reported by 45% of participants. After treatment using soft tissue manual therapies combined with education and training, most participants reported large or moderate reductions in pain severity. This holistic interdisciplinary approach provides practical support, trust building, listening therapies, education, and non-invasive therapeutic approaches	Pain Severity
Hasha et al [18].	Norway	Syrian	101	To evaluate the effect of group physiotherapy and awareness intervention to reduce pain disorders, and secondarily improving mental health, in Syrian refugees	Quantitative – Randomized Controlled Trial	The physiotherapy group intervention (PAAI) had no effect on reducing either pain disorders or improving mental health in Syrian refugees living in Norway	Sociocultural Considerations; Access to Adequate Healthcare and Medication; Psychological Correlates; Pain Severity
Gammoh et al [19].	Jordan	Syrian	384	To screen for fibromyalgia (FM), characterized by chronic widespread pain, fatigue and sleep disturbances, and insomnia prevalence and severity. To study the correlation between FM severity and insomnia. To study FM treatment trends among female Syrian refugees in Jordan	Quantitative – Cross-sectional Questionnaires	High frequency of moderate and severe subjective FM among women refugees and showed correlations with insomnia. 60% of the sample were using Acetaminophen treatment. Current treatments are suboptimal, requiring earlier screening and raising awareness of FM diagnosis and treatments is needed	Psychosocial Correlates; Integrative Pain Management Interventions; Pain Severity
Berthold et al [20].	United States	Cambodian	186	To examine the role of communication with a health care provider as a predictor of self-reported pain severity among Cambodian American refugees	Quantitative – Secondary Survey Data	Greater difficulty understanding the healthcare provider, depressive symptoms, trauma symptoms, food insecurity, and social isolation, were all predictors of higher self-reported pain scores. Beyond all other social determinants, difficulty communicating with the healthcare provider and depressive symptoms were uniquely associated with greater self-reported pain, suggesting insufficient access to detailed clinical pain assessments	Pain Severity; Access to Adequate Healthcare and Medication; Sociocultural Considerations; Psychological Correlates
Strømme et al. [21]	Lebanon & Norway	Syrian	353	To examine chronic pain, migration-related exposures, and mental health among Syrian refugees transiting from Lebanon to Norway. These factors were assessed at baseline and follow-up, 1 year after arrival in Norway, to investigate temporal changes in the relationship, treatment, and predictors of chronic pain and mental health problems	Quantitative – Prospective longitudinal study using Questionnaires and Structured Telephone Interviews	The study reported a greater association between migration-related stressors, chronic pain, and mental health problems after resettlement in comparison to the transit phase. Whilst poor mental health was not associated with chronic pain at baseline, it was a statistically significant predictor of chronic pain at follow-up among refugees who did not report chronic pain at baseline, suggesting a potential direction of causality between mental health problems and pain. At both time points, one in four participants with chronic pain used analgesics	Integrative Pain Management Interventions; Psychological Correlates; Access to Adequate Healthcare and Medication
Nissen et al [22].	Norway	Syrian	902	To examine the prevalence of chronic pain among Syrian refugees in Norway; to investigate the association between chronic pain and mental health problems within this population; and explore how chronic pain and mental health problems are related to perceived general health and functional impairment	Quantitative – Cross-sectional Study using Questionnaires	Within the study population, the study shows a high burden of chronic pain, with 43.1% reporting severe chronic pain, who likely experience functional impairment. There was strong evidence that higher levels of chronic pain were associated with mental health problems, specifically anxiety, depression, and PTSD. Therefore, the prevalent co-occurrence of both problems suggests multidisciplinary treatment may need to be adopted to be able to effectively mitigate either problem	Pain Severity; Psychological Correlates; Integrative Pain Management Interventions; Access to Adequate Healthcare and Medication
Al-Smadi et al [23].	Jordan	Iraqi and Syrian	288	Assess the impact of fibromyalgia (FM) and its associated factors in female refugees	Quantitative – Cross-sectional Study using Questionnaires	73.62% of participants reported moderate to severe FM impact. Increased age, anxiety, PTSD, living in Irbid and being Iraqi were predictors of higher FM impact	Pain Severity; Psychological Correlates; Integrative Pain Management Interventions; Access to Adequate Healthcare and Medication
Demirtas et al [24].	Turkey	Includes: Syrian, Iraqi, and Afghan	278	To determine the prevalence of chronic pelvic pain (CPP) in refugee women and examine associated factors and its impact on quality of life	Quantitative – Cross-sectional Study using Questionnaires	CPP prevalence was significantly higher in refugee women (41%) than non-refugee women (19.1%). Refugee status, low income, anxiety, low back pain, dyspareunia, three or more miscarriages, history of gynaecological surgery, diarrhea, and urinary tract infections were identified as risk factors for developing CPP	Pain Severity; Sociocultural Considerations; Psychological Correlates; Access to Adequate Healthcare and Medication
Harlacher et al [25].	Cambodia	Cambodian	113	To investigate the effectiveness of pain psychoeducation for chronic pain in trauma survivors	Quantitative – Randomized Controlled Trial	Limited evidence suggests a positive impact of psychoeducation on pain-related outcomes. The study found a significant within-group effect for pain intensity reduction from pre- to post-treatment in the treatment group (pain psychoeducation group)	Pain Severity; Access to Adequate Healthcare and Medication; Integrative Pain Management Interventions
Dadras & Diaz [26]	Norway	Syrian	154	To investigate the association of perceived discrimination with self-rated health, chronic pain, mental health, and healthcare use among Syrian refugees	Quantitative – Cross-sectional Study using Surveys	Participants reported high levels of perceived discrimination. Those who have experienced discrimination were more likely to suffer from mental health symptoms. The study found no relationship between perceived discrimination and chronic pain	Pain Severity; Sociocultural Considerations; Psychological Correlates
Standnes et al [27].	Norway	Syrian	353	To explore associations between pain level and use of painkillers, healthcare services, and long-term impairment among Syrian refugees	Quantitative – Cross-sectional Study using Surveys	27% of Syrian refugees reported chronic pain 1 year after resettlement. Levels of pain were high for their age compared to the general population. High pain levels were associated with increased use of painkillers, healthcare services and long-term impairment. Trauma experiences were associated with chronic pain	Pain Severity; Access to Adequate Healthcare and Medication; Integrative Pain Management Interventions
Rometsch et al [28].	Germany	Northern Iraqi	116	To analyze symptoms of pain, other somatic complaints, and concepts of illness in traumatized female refugees	Mixed Method—Interviews	Pain is the main somatic complaint with a moderate rated severity, followed by feelings of suffocation and movement disorders. Somatic symptoms are mainly attributed to psychological causes, following physical, religious, and supernatural causes. Women with pain symptoms more to physical causes than women without pain symptoms	Pain Severity; Sociocultural Considerations; Psychological Correlates
Campeau [29]	United States	Somalia	15	To examine how refugees at a Somali refugee women’s health center interact with and change medical knowledge and practices in inventive and constrained ways	Qualitative – Interviews	Identified four emerging frameworks for which female Somali refugees experience chronic pain: (1) pain as a symptom of exile, (2) pain and the strength to bear pain as issues of faith, (3) medicine as powerful, curative and fluid, (4) medical discrimination and exclusion. These frameworks highlight the social and cultural context to their experience of pain, expectations, hope, and management strategies	Sociocultural Considerations; Access to Adequate Healthcare and Medication
Park et al [30].	South Korea	North Korean	20	To examine different understandings of chronic pain between North Korean Refugee women and the South Korean healthcare providers. To identify strategies to mitigate these differences and improve pain management	Qualitative – Semi-structured Interviews	Four themes were identified: characteristics of physical pain experienced; physical factors as primary causes of pain; psychological factors as collateral causes of pain; and pain management experiences and care expectations. North Korean refugee women seemed to view their pain as physical whilst healthcare providers in South Korea interpreted their pain as psychological. This chasm of perception may be a barrier to healthcare utilization and treatment adherence	Access to Adequate Healthcare and Medication; Psychological Correlates; Integrative Pain Management Interventions; Sociocultural Considerations
Müllersdorf et al [31].	Sweden	Middle Eastern	10	To explore the meaning of reciprocal recognition for women from the Middle East experiencing chronic pain after forced resettlement	Qualitative – Semi-structured Interviews	This study highlights the importance of understanding the impact of trauma and forced displacement on chronic pain experiences. Factors contributing to a good meeting included: time, dialogue, understanding, and honesty. Not being properly examined, not being offered optimal treatment, and not being believed were signs of dismissal. Conclusions suggest reciprocal recognition and support connected to the experiences of patients are vital to achieve a holistic approach to pain management	Access to Adequate Healthcare and Medication; Integrative Pain Management Interventions
Altun et al [32].	Australia	N/A	N/A	Explore the experiences of GPs providing care for refugee women with chronic pain	Qualitative – Semi-structured Interviews	GPs acknowledged the unique needs for refugee women but were challenged by the complex consultations; system-level issues like time, funding and resource constraints created significant challenges treating chronic pain. They highlighted the importance of cultivating culturally safe clinical environments and hearing patient stories	Access to Adequate Healthcare and Medication; Sociocultural Considerations; Integrative Pain Management Interventions
Altun et al. [33]	Australia	Assyrian Iraqi	10	To explore the experiences of Assyrian refugee women seeking care for chronic pain	Qualitative – Semi-structured Interviews	Interviews with women identified five major themes regarding their experiences accessing care for chronic pain. Refugee women arrive in Australia with a host of needs, to which are seldom seen to due to affordability, language fluency, and health system literacy. Women in particularly balance competing priorities such as family welfare. This study suggests provision of interpreters and employing a bottom-up approach to develop strategies to improve access to care	Access to Adequate Healthcare and Medication; Sociocultural Considerations
Nigol et al [34].	Australia	Bhutanese	22	To explore conceptualizations of chronic pain among Nepali-speaking Bhutanese adults	Qualitative – Focus Group Interviews	Nepali-speaking Bhutanese people conceptualized their chronic pain as persistent, subjective and poorly understood. Participants believed pain to be complex and multifaced, requiring active and passive strategies for management, which are also culturally informed. Of note, participants needed healthcare provider appreciation for cultural humility, historical trauma, and their pain story	Sociocultural Considerations; Integrative Pain Management Interventions
Zander et al [35].	Sweden	N/A	N/A	To explore 35 healthcare providers’ perceptions of causes of chronic pain and gathering suggestions for rehabilitation for women from the Middle East	Qualitative – Questionnaires	Healthcare professionals perceived chronic pain in these women as complex and multifaceted, primarily influenced by psychosocial factors, such as experiences of grief, trauma, and loss. Culture clashes and conflicting priorities of family and work were perceived as affecting pain. The study participants recommend greater education about pain and pain management for these women	Psychological Correlates; Access to Adequate Healthcare and Medication; Sociocultural Considerations; Integrative Pain Management Interventions
Brodda Jansen [36]	N/A	Somalian and Iraqi	2	To illustrate the important areas that need to be addressed within the health care system to improve persistent pain in refugees	Case report	There is a need to improve knowledge about pain in refugee women in the healthcare system. Treatment must focus on biopsychosocial efforts. Education about pain and trauma should be mandatory	Pain Severity; Psychological Correlates; Access to Adequate Healthcare and Medication
Longacre et al [37].	N/A	N/A	N/A	To examine the efficacy and feasibility of complementary and alternative medicine modalities to improve pain with respect to survivors of torture and refugees with trauma	Review – Literature Review	Preliminary research suggests that complementary and alternative modalities including reiki, chiropractic, acupuncture, yoga and physical practice, meditation, pranayama/yogic breathing, and massage/body work are promising treatment approaches for chronic pain, though research is limited	Pain Severity
Altun et al [11].	N/A	N/A	N/A	To evaluate the effectiveness of interventions for chronic pain management when applied in the context of refugee and immigrant populations	Review – Systematic Review	21 studies were included. Most interventions involved multidisciplinary or psychological interventions. Studies that included multidisciplinary approaches to management reported more consistent improvements in pain intensity and functional improvements than unimodal approaches	Pain Severity; Psychological Correlates; Sociocultural Considerations; Integrative Pain Management Interventions; Access to Adequate Healthcare and Medication
Bang et al [38].	United States	Southeast Asia	N/A	To integrate the current findings on the health of Southeast Asian refugees resettled in the United States into a review	Review – Integrative Review	Southeast Asian refugees experienced worse physical and mental health than the general population in the United States, with chronic pain (8%−51% reported chronic pain in the included studies) as one of the most common physical health problems. Experiences of trauma, discrimination, and comorbid mental health problems were reportedly associated with poor physical health and engagement with healthcare services. Significant barriers to receiving healthcare were language differences, transportation, and lack of health insurance	Pain Severity; Access of Adequate Healthcare and Medication; Psychological Correlates; Sociocultural Considerations
de C Williams & Baird [39]	N/A	N/A	N/A	To review the recent evidence informing the assessment and treatment of pain in survivors of torture and war, with a broader context of psychological distress outlining the clinical and research implications	Review – Clinical Review	Pain management in most parts of the world is poorly resourced and absent from the public health agenda. Clinicians must ask and listen to the experiences. Medical colleagues continue to dismiss chronic pain when there are no physical signs or where there are signs of psychological distress	Pain Severity; Psychological Correlates; Access to Adequate Healthcare and Medication
Rometsch-Ogioun El Sount et al [40].	N/A	N/A	N/A	To summarize existing evidence about patient characteristics and specific interventions targeting pain in traumatized refugees and to give an overview of chronic pain symptoms in refugees with PTSD	Review—Systematic Review	Pain symptoms were associated with older age, female gender, general living difficulties and PTSD symptoms. CBT and NET with biofeedback, manualized trauma psychotherapy, TCM and Emotional Freedom Techniques were evaluated as specific interventions, resulting in positive outcomes for both pain severity and PTSD symptoms	Pain Severity; Psychological Correlates; Sociocultural Considerations
Crosby [41]	N/A	N/A	N/A	To discuss the methods for obtaining refugee trauma histories, recognize the psychological and physical manifestations of trauma characteristics and explore how culture differences and limited English proficiency affect the refugee patient-clinician relationship and how to best use interpreters	Review—Clinical Review	Engagement with a refugee patient who experienced trauma requires an understanding of the trauma history and the trauma-related symptoms. Mental health symptoms and chronic pain are commonly experienced. Successful treatment requires a multidisciplinary approach that is culturally acceptable to the refugee	Sociocultural Considerations; Access to Adequate Healthcare and Medication; Psychological Correlates
Lies et al [42].	N/A	N/A	N/A	To review the epidemiology and evidence-based management of PTSD, sleep disturbance, and pain in refugees	Review—Narrative Review	Epidemiological evidence shows that more than 50% of refugees suffer from sleep disturbance, pain and PTSD. This review highlights the need for culturally sensitive treatment strategies that consider language barriers, expression of distress, loss of freedom, and threat of repatriation. It also notes the high prevalence of pain among refugees with comorbid PTSD. Psychoeducation, self-managing skills development, manual therapy, opioids, and NSAIDs have been shown to be effective for treating pain	Pain Severity; Sociocultural Considerations; Psychological Correlates; Integrative Pain Management Interventions

^*^Refugees and asylum seekers

^**^Only top 5 nationalities were reported

#### Quantitative studies

Of the twelve quantitative [9, 17–27] studies, and one quantitative section of a mixed methods study [28], seven studies adopted a cross-sectional design [19, 22–24, 26–28] and two were secondary data analyses [9, 20]. Other study designs included a longitudinal prospective cohort study [21], a case study [17], and two randomized controlled trials [18, 25]. Of the thirteen included studies, five only investigated female participants [9, 19, 23, 24, 28] and eight investigated a sample of both genders [17, 18, 20–22, 25–27]. Study details can be found in Table 1.

#### Qualitative studies

Of the seven qualitative studies [29–35] and one qualitative part of a mixed methods study [28], the most common form of data collection was individual interviews [28–33], with observations [29, 30], focus groups [28, 34] and Questionnaires [35] also adopted. Of the eight studies, four investigated a female only sample [29–31, 33], and four investigated a sample of both genders [28, 32, 34, 35], of which two studies investigated the perspectives of healthcare professionals regarding the management of chronic pain in female refugees [32, 35]. Study details can be found in Table 1.

#### Reviews

Of the review studies included, two were systematic reviews [11, 40], and two studies were clinical reviews [39, 41]. Other review designs included an integrative review [38], a case report [36] a literature review [37] and a narrative review [42]. The two systematic [11, 40] and one integrative review [38] reported including between 15 and 21 studies in their analyses. Further details in Table 1.

#### General rhemes

##### Pain severity

The theme pain severity reflects a critical area of study among refugees. Greater pain severity was observed among refugee populations compared to compatriot patients [27, 38] and greater prevalence than the comparative host country population [24, 38]. In one study, the prevalence of Chronic Pelvic Pain (CPP) in women was 1.68 times higher in the refugee group than in the non-refugee group [24]. Chronic pain was self-reported to be present for at least 12 months to several years [9, 17, 27]. A study of Syrian refugees in Norway found that over 25% reported chronic pain one year after resettlement [27].

Chronic pain was strongly associated with long-term disability, functional impairment, and poor perceived general health [9, 22, 27]. Higher levels of pain were also associated with increased use of non-prescription and prescription painkillers and increased use of emergency rooms, specialist care, and hospitalization [27]. However, limited research exists on effective treatments for pain in refugee populations [42]. Treatments used for moderate to severe acute and chronic pain in refugees include: opioids (e.g., codeine, tramadol) and non-steroidal anti-inflammatory drugs (NSAIDs) (e.g., ibuprofen, paracetamol, metamizole) [42].

Moreover, correlations have been drawn between pain severity and increased odds of chronic pain with gender (with women often experiencing greater severity and risk) [9, 22]. Particularly amongst women, trauma exposure was associated with chronic pain, with one study finding that women experiencing 1–2 traumas before migrating were 3.3 times more likely to have chronic pain and long-term disability than women with no history of trauma [9, 21]. Healthcare professionals perceive chronic pain in refugee women as complex, often influenced by trauma, grief, and loss [32, 35].

### Psychological correlates of chronic pain

The literature highlights the relationship between psychological conditions and chronic pain among refugee populations, emerging as either a primary or secondary focus in most studies, indicating its substantial influence on pain experiences, intensity, and duration [9, 19–24, 26, 28, 29, 38–41]. A well-established finding pertains to the bi-directional relationship between psychological factors and chronic pain, suggesting a complex interplay wherein each exacerbates the other [9, 19, 21, 22, 28, 40]. Specifically, post-traumatic stress disorder (PTSD), anxiety, and depression emerge as significant correlates of chronic pain among refugees [20, 22, 23, 40, 42]. Interestingly, some studies suggest that symptoms of these mental health problems were predictors of chronic pain, perhaps a result of resistant mental health challenges having a greater vulnerability to chronic pain [20, 21, 23, 32]. Two studies suggested that the concurrence of psychological illness and chronic pain may be explained by mutual maintenance theory [11, 21]. Preliminary evidence suggests that psychological interventions, specifically cognitive behavioral therapy and narrative exposure therapy, were highly effective in refugees with comorbid PTSD and chronic pain [40]. Alternatively, Altun and colleagues’ systematic review (2022) [11] suggested that psychological interventions were more effective when grounded in a biopsychosocial model of care. Campeau (2018) reported that Somali refugee participants supported this notion, with feelings of frustration when their pain was attributed only to the psychological, psychosomatic, or psychogenetic causes [29]. Therefore, as advocated by most included studies, one’s mental and physical health cannot be treated in isolation and treatment plans assigning pain to only psychological causes should be avoided [32, 39].

#### Barriers to optimal pain management among refugees

##### Access to adequate healthcare and medication

Adequate and timely treatment of acute pain reduces the likelihood of developing chronic pain [39]. Therefore, accessing pain relief promptly is a priority. Regular pharmacological treatments are vulnerable to disruptions within this population, however after resettlement in a host country, refugees continued to take fewer analgesics than the corresponding host population [21]. This may be a result of cultural deterrents to frequent medication use or represents a barrier to medication accessibility [32]. In one case study, pain prescription pills were unused because the instructions were written in English [41]. Misunderstandings or miscommunication due to language barriers or limited literacy levels exacerbate the difficulties in accessing and navigating healthcare services [9, 12, 29, 33, 38]. Beyond accessibility, higher self-reported difficulty understanding the healthcare provider predicted higher self-reported pain scores, which may be a result of ineffective pain assessment, diagnosis, or management [20, 41]. Both physicians and patients reportedly become frustrated explaining and understanding chronic pain symptoms, resulting in negative experiences in the healthcare system [36, 41]. The nuanced understanding and expression of "pain" across different languages and cultures further complicates communication between refugees and healthcare providers [22]. As such, different understandings of chronic pain can be a barrier to healthcare utilization, even when sharing language [30].

Logistic hurdles, such as residential status, transportation difficulties, and resettlement in rural areas impede refugees' ability to seek timely and appropriate care [9, 29, 32, 34, 38, 40]. Financial constraints exacerbate healthcare disparities as many refugees are unable to engage in paid employment, lack health insurance, and have limited financial support, particularly for women who may be economically dependent on male figures [38].. In most host countries, pain management is poorly resourced [39]. Basic needs such as food security, safety, and adequate living conditions often take precedence over healthcare concerns, relegating pain management to a lower priority or rendering it unattainable in survival-focused contexts [33]. Thus, resources need to be shifted to refugee populations to provide equitable and accessible healthcare [39].

Among these challenges is the pervasive sense of distrust and unfamiliarity with Western healthcare systems experienced by many refugees [38]. This distrust can stem from current circumstances in their home country, past negative experiences, cultural differences, or a lack of understanding of the healthcare system's structure and processes [29, 38]. Medical treatment for pain offers a limited and slow-moving resource for refugees, being described as ‘complicated’ and ‘exhausting’, leading many of them to seek care only if reactions were severe [29]. Instead, refugees in one study adopted more accessible and regular forms of care, such as complementary or faith-based medicine [29].

It is reported that female refugees face additional barriers in accessing optimal pain management. Sexual and gender-based violence, primarily against women, can ostracize them from their families and communities, disconnecting women from their social support networks [41]. Furthermore, women with chronic pain are more likely to have their pain attributed to psychiatric causes, adding to their limited access to optimal pain management [11].

##### Integrative pain management interventions

A holistic approach to pain management that considers the complex interplay of cultural and psychological factors is crucial for effective treatment of chronic pain in refugee populations. The prevalence of unimodal interventions emerges as a significant barrier to optimal pain management among refugee populations, as highlighted by several key observations in the literature [11, 18, 36]. This narrow focus can limit the long-term effectiveness of interventions, particularly in addressing the multifaceted nature of chronic pain experienced by refugees [11, 25, 32]. This is particularly evident in the limited treatment options for comorbid PTSD and pain, in the general and refugee populations [28, 39, 42]. Moreover, there are reported inconsistencies between the therapies or medications prescribed and established guidelines for pain management [19].

Successful multidisciplinary interventions often integrate psychological therapies, physical therapy, and self-management strategies that empower refugees to actively participate in their care [25, 32]. Whilst psychological therapies, like Cognitive Behavioral Therapy (CBT), mindfulness-based stress reduction, and trauma-focused therapies, help refugees develop coping mechanisms for pain and stress, manage anxiety and depression, and reduce the emotional distress that often exacerbates pain perception [32, 42], physical therapy is crucial for improving mobility, strength, and function in refugees experiencing chronic pain [25, 32]. Interventions that incorporate self-management techniques are also recommended as part of a multidisciplinary pain management plan [32, 42]. One such intervention, pain psychoeducation, has shown preliminary evidence of effectiveness in reducing pain intensity [25].

Trauma, particularly PTSD, is a frequent and complicating factor in the management of chronic pain [23, 32]. Given the prevalence of trauma experiences and comorbid PTSD in this population [22, 41, 42], integrating trauma-informed care is crucial for effectively addressing chronic pain in refugees. Trauma-informed care acknowledges the impact of trauma on an individual's life and how it affects their engagement with healthcare services, emphasizing the importance of creating a safe and trusting environment for patients to share their experiences [32]. One way of achieving is through reciprocal recognition and support connected to the specific experiences of patients [31].

Community-based interventions can be effective in reaching refugees who may face barriers to accessing traditional healthcare settings [32]. These interventions can include outreach programs, support groups led by trained community members, and educational workshops that provide information about pain management, coping skills, and available resources. Partnering with community organizations that serve refugee populations and engaging refugee leaders in program design can ensure cultural appropriateness and improve program accessibility [32].

Complementary and alternative medicine may be a culturally appropriate and effective part of an integrated treatment plan that is more accessible to refugee populations. Providing alternative modality clinics may provide refugees with a more accessible and comprehensible form of care to use alongside Western orthodox medicine [37]. Recommendations include exercise, meditation, soft tissue manual therapies, psychological therapies, and facilitating social connection [11, 17, 18, 42]. Many of these management tools may be applied at home which reduces dependence on pain medication [11]. As there is no sure-fire pain management approach for success, integrating multiple disciplines with a focus on holistic refugee rehabilitation and well-being can create opportunities and potentialities for success [17].

##### Sociocultural considerations

The profound influence of cultural norms, traditional beliefs, and community support systems on how refugees experience and manage chronic pain is evident in the literature. Both pre- and post-migration factors were associated with self-reported chronic pain [9, 24, 33]. Pre-migration experiences of trauma, violence, torture and displacement are central to refugees’ chronic pain and can be exacerbated by post- and peri-migration factors [9]. Post-migration stressors included unfamiliar jobs or unemployment, financial hardships, lack of social support, discrimination and difficulty integrating into a new environment [22, 24–27, 32–35, 38, 42]. When there is a cultural disparity between refugees and healthcare providers, this poses a significant challenge to chronic pain management and may hinder effective healthcare delivery [32–34, 41]. Different understandings of illness and healthcare present challenges to interpreting pain and its causes, particularly when co-occurring with psychological illness [30, 32, 33, 35, 38, 42]. Without a clear medical narrative for chronic pain, many refugees interpreted their pain in relation to their pre- and peri-migration experiences [29]. Additionally, refugees might interpret pain as a spiritual consequence or rely on traditional healing practices that differ from Western medicine [33, 34, 42]. A biopsychosocial approach considers cultural nuances to some extent, however understanding an individual’s trauma, personal, and cultural history and familiarity with cultural health practices is critical for treating this population [36, 41].

Refugees frequently experience discrimination in their host country. With the exception of Dadras and Diaz’s non-significant results [26], discrimination has been associated with chronic pain and suggested to be a predictor of PTSD and depressive symptoms [9, 24, 33, 38, 41]. Additionally, discrimination was commonly reported in settings of social support, work, and within neighborhoods, which may act as a barrier to accessing healthcare services [9, 33]. However, relationships forged in refugee communities serve as a source of support and engagement in social groups was associated with better self-rated health and health profiles [9, 18, 38]. Strong family and community support can act as a buffer against these negative impacts, providing emotional resilience and practical assistance in navigating challenges [33, 34].

Cultural and societal norms can shape the experience and expression of pain differently for men and women [33, 34]. Refugee women often prioritize family welfare over seeking help for their pain, which can lead to medical neglect [33]. General practitioners (GPs) in Australia and Sweden reported that consultations with refugee women with chronic pain were often complex due to the multifaceted nature of chronic pain, health system-level issues, and culture clashes, conflicting priorities of family and work [32, 35].

Faith and religion could function as a positive or negative influence on coping with chronic pain [28, 40]. For some, religious beliefs served as sources of solace and hope, however for others, religious participation was related to higher levels of psychological distress, perhaps due to histories of ethnic-related trauma [29, 38]. The preference for alternative and faith-based treatments persists among refugee populations, reflecting both cultural traditions and dissatisfaction with Western medical approaches [38]. Alternative and homeopathic management methods included prayer, meditation, acupuncture and massage [29, 38].

### Identified gaps in research and practice

Despite the high prevalence of chronic pain in refugee populations, research specifically focusing on this issue remains limited. There is a scarcity of high-quality empirical studies evaluating the effectiveness of pain management interventions in this population. Many existing studies are descriptive rather than interventional. Two randomized controlled studies investigated the effectiveness of alternative pain treatments in reducing pain disorders, but the results were limited [25] or non-significant [18]. Generally, there is a lack of literature on the treatment of pain compared to psychological strategies. Research and clinical practice often fail to address the long-term impact of chronic pain on refugees' physical and mental health, social integration, and overall well-being [27]. As such, longitudinal studies are needed to understand the trajectory of chronic pain in refugee populations and to develop interventions that promote long-term health and quality of life. Reflecting on practice, many medical professionals may dismiss chronic pain where psychological distress is present [39]. Many healthcare providers lack sufficient training and knowledge regarding the specific healthcare needs of refugees, including chronic pain management [32]. Additionally, Existing community resources and support networks that could benefit refugees with chronic pain are often underutilized [34].

### Summary of findings

Narrative synthesis of the included studies reveals systemic issues in humanitarian prioritization of healthcare needs for refugees. Refugees experience a high prevalence of chronic pain, with research indicating a rate significantly exceeding that of non-refugee populations and host countries. The research highlights that this disparity stems from a complex interplay of pre-migration trauma, including violence, torture, and displacement, compounded by post-migration stressors such as discrimination, economic hardship, and social isolation. Chronic pain in refugees carries serious implications, contributing to long-term disability, functional impairment, and diminished overall health, often leading to increased reliance on healthcare services and pain medication. Notably, the research emphasizes the strong correlation between mental health and chronic pain in refugee populations, underscoring the need for comprehensive treatment approaches that address both physical and psychological aspects of pain. The research identifies barriers hindering refugees' access to effective pain management, encompassing sociocultural disparities in healthcare perceptions, limited healthcare access due to language differences and financial constraints, and gender-specific challenges faced by refugee women. To mitigate these obstacles, the sources advocate for a holistic, biopsychosocial model of care that considers the multifaceted nature of chronic pain in refugees and incorporates interventions such as multidisciplinary treatment, trauma-informed care, community-based programs, and complementary therapies. Key gaps in the knowledge base relate to limited interventional studies evaluating multidisciplinary culturally appropriate chronic pain management strategies and the long-term outcomes for refugees.

## Discussion

This scoping review identified 28 articles on chronic pain and its treatment in refugees. The field suffers from a dearth of interventional research, limited control groups, reliance on unstandardized self-reported measures of pain, and short follow-up periods, showing little improvement over time. It appears there is a greater focus on mental health, psychological interventions, and the repercussions of PTSD, at the expense of chronic pain issues prevalent among refugee populations. There is an urgent need for a shift in focus towards prioritizing empirical longitudinal research to address the complex challenges of pain management in refugee communities.

Engagement with healthcare services is vital for managing pain in refugee populations. Significant challenges refugees face in accessing healthcare services include affordability, geographic accessibility, and service quality [9, 29, 31–34, 38, 40]. Participants described healthcare systems as convoluted and difficult to navigate, with barriers exacerbated by structural inequalities based on race, ethnicity, gender, and socioeconomic status [29]. Despite acknowledging these barriers, few studies address the underlying factors hindering refugee engagement with healthcare. It is imperative for healthcare planners and clinicians to proactively address these barriers to ensure continuous care for refugees. Furthermore, there exists a tendency within healthcare provision to treat refugees as a homogeneous group, overlooking the diversity of experiences and symptoms, particularly in categorizing refugees with torture survivors [43]. This homogenization can hinder effective care and recovery outcomes for these vulnerable populations [44].

Language, literacy and cultural linguistic norms present additional challenges to treating chronic pain. Communication difficulties are among the most significant impediments to receiving healthcare, leading to misdiagnoses and hindering the patients’ ability to actively participate in their own care [20, 38]. Expressing chronic pain is inherently challenging, and this burden is magnified for individuals navigating language and literacy barriers. Complicating matters, invisible injuries contributing to chronic pain can exacerbate feelings of frustration and overwhelm both patients and healthcare professionals [45]. Whilst this invisibility contributes to feelings of isolation for patients as they struggle for recognition and validation of their suffering [46], healthcare professionals may feel frustrated when they cannot adequately address the complexities of chronic pain [47]. Together, the patient-provider relationship may be strained. Perceptions and beliefs have a direct influence on an individuals’ response to illness and adherence to treatment, acting as both a facilitator and barrier [48]. Therefore, for patients who perceive their pain to be physical, their care expectations would be focused on physical treatment, implicated to be a more effective approach [30].

Sociocultural factors influence the manifestation of psychosomatic symptoms and shape coping mechanisms in refugee populations, through their religion, culture, and collective identity. Pain is construed differently across cultures, and misalignments in perceptions of pain between patients and healthcare professionals can lead to misdiagnoses and ineffective treatment, as seen in research comparing North Korean refugee women’s pain perceptions with healthcare professionals’ interpretations [30]. Such discordances are more evident when comparing Western and Eastern cultures. For instance, terms used to describe psychological distress may not directly translate across languages, leading to misinterpretations and misdiagnoses. For example, demoralization may be mislabeled as depression, and the direct translation of “depression” in Somali, “wal-wal” means “crazy” [49].

Additionally, cultural norms, such as those prohibiting physical contact between genders, must be respected to foster trust and facilitate effective care [41]. Lagisetty and colleagues [50] proposed interventions could be more effective following a framework of cultural sensitivity, targeting all four domains: facilitator, location, language, and messaging (FiLLM). Given these complexities, it is recommended for healthcare providers to receive education specific to treating refugee populations and to be sensitive to cultural variations in customs [42].

Refugee women face unique challenges in seeking and receiving care for chronic pain [24]. Traditional gender roles and cultural norms can influence their help-seeking behaviors, leading to underreporting of pain or delays in seeking medical attention [33, 34]. In some cultures, women are expected to prioritize the needs of their families over their own health, potentially leading to neglect of their pain conditions [33]. Demirtaş et al. (2024) [24] highlight a concerning finding: refugee women are at a significantly higher risk of developing chronic pelvic pain compared to non-refugee women. It's crucial to understand the underlying reasons for this increased risk, which might include factors like pre-migration experiences of sexual violence, inadequate access to reproductive healthcare, or cultural stigma surrounding pelvic pain [24]. Furthermore, healthcare providers should be aware of these gender-specific challenges and create a safe and supportive environment where refugee women feel comfortable discussing their pain and seeking help.

The consequences of inadequate chronic pain management for refugees have far-reaching implications for individuals’ well-being and their coping strategies, perpetuated by feelings of blame towards healthcare providers [29, 51]. Such negative emotional states can act as significant barriers to healthcare utilization. Consequently, some studies report an increased reliance on faith and religion for solace and healing [29]. However, participants have highlighted the dichotomy between pursuing medical interventions and turning to faith-based understandings of illness, reflecting flexible fatalism, wherein individuals recognize the limitations of medical interventions but do not view biomedical and religious approaches to health as mutually exclusive [29]. Therefore, fatalism may serve as a strategic and pragmatic coping mechanism for navigating a healthcare system fraught with financial, logistical, and language barriers.

Chronic pain poses a complex clinical challenge in refugee populations, due to its diverse etiologies and compounded by psychosocial stressors inherent in the resettlement process. Studies show that these stressors and barriers exacerbate health issues, including chronic pain [52]. Among refugees, PTSD emerges as the most prevalent mental health diagnosis, with rates ranging from 31 to 87% [53, 54]. Studies have shown that the presence of PTSD can significantly worsen pain outcomes in refugees [55]. In a study by Nordin and Perrin (2019) [55], pain interference, as opposed to pain severity alone, was found to be a stronger predictor of poorer PTSD treatment outcomes in tortured and traumatized refugees. Recognizing the high comorbidity between chronic pain and PTSD highlights the need for a comprehensive interdisciplinary approach to effectively address both conditions [21, 22]. Mutual maintenance theory elucidates how chronic pain and PTSD perpetuate each other, highlighting the interplay between physical and psychological distress [11]. For example, the hypervigilance and emotional dysregulation associated with PTSD can intensify pain sensations, potentially making it harder to engage in coping mechanisms [55]. Healthcare planners and clinicians must acknowledge these interrelations to develop tailored interventions for refugees. However, chronic pain is often inadequately recognized within refugee populations and may, in fact, be overshadowed by disorders such as PTSD [56]. This oversight can result in chronic pain being misconstrued as a non-specific symptom of psychological disturbance, resulting in missed diagnoses and long-term complications [57, 58]. In this regard, psychological interventions alone have shown inconsistent outcomes, underscoring the inadequacy of a purely psychological approach to chronic pain management.

Chronic pain management for survivors of conflict presents unique challenges, particularly where specialized care is limited. Non-specialist medical staff often bear the primary responsibility for managing chronic pain among these populations, relying on simple standardized protocols for pain assessment and treatment, especially in surgical projects (e.g., using the Médecins Sans Frontières Clinical Guidelines [59]). However, in cases involving complex reconstructive surgery and rehabilitation, a more specialized approach to pain management becomes imperative [60]. Survivors of conflict undergoing reconstructive surgery frequently experience significant perioperative pain, which may be further compounded by pre-existing pain conditions [51]. Moreover, some patients may have developed opioid dependence, necessitating innovative approaches to pain management [47, 51, 58]. Regional anesthesia techniques, adjunctive agents, and opioid-sparing strategies are increasingly recognized as vital components of comprehensive pain management protocols for this vulnerable population.

Insufficient expertise of chronic pain physiology, coupled with a lack of emphasis on medical assessment and limited knowledge of available treatment options, challenges the effective management of chronic pain within refugee populations [36, 61]. Healthcare providers may deviate from recommended guidelines [19], leading to disparities in treatment outcomes and potentially compromising the quality of care received by refugees. A blind-comparison study conducted by Kaur et al [55] using the United Nations Istanbul Protocol (UNIP), revealed that a significant portion of pain remains undetected due to the absence of evidence-based diagnostic tools and the presence of confounding psychiatric illnesses. This leads to misdiagnoses and the treatment of pain solely as a manifestation of psychological trauma, neglecting its multifaceted nature [62]. Furthermore, an overemphasis on psychological consequences and mental treatment interventions, coupled with barriers related to social, cultural, and linguistic components, further impedes refugees’ access to appropriate pain management interventions.

Multidisciplinary approaches to chronic pain management, grounded in the biopsychosocial model, have garnered significant attention within both clinical practice and research domains. This model recognizes the interplay of biological, psychological, and social factors in shaping pain experiences [63, 64]. Whilst multimodal treatments for chronic pain may not be necessary for all patient scenarios, a biopsychosocial approach is needed for long-term benefits [11]. The lack of such approaches presents a barrier to optimal pain management in refugee populations, specifically for comorbid chronic pain and PTSD [39, 65]. Although some included studies reported a patient preference for physical treatments [20, 28, 30], providers tended to recommend non-physical treatment [28, 30], which may be grounded in the current research that focuses primarily on mental health problems within this population. As such, a mismatch exists between the burden of chronic pain in refugee populations and the available evidence to guide pain management strategies. Moving forward, it’s crucial to extend the focus into physical, cultural, and social health of refugees to provide approaches acceptable to the patients being treated.

### Limitations

Several limitations warrant consideration when interpreting this scoping review’s findings. Firstly, the relatively small number of selected articles, including reviews, may reflect the current state of the literature but also raises the possibility of inadvertently excluding relevant articles. The scarcity of appropriate studies represents a notable limitation for this review and its future applicability. Additionally, restricting inclusion to English-language articles may omit valuable information available in other languages. Access issues could have further limited article inclusion. Another challenge is the variability of measurement tools used to assess self-reported pain severity, impeding direct study comparisons. Future research should prioritize harmonizing and standardizing methodologies for pain measurement to facilitate meaningful comparisons and enhance the robustness of findings. The authors acknowledge the lack of a-priori protocol registration as a limitation of this study, which may introduce potential biases in study design and analysis. Finally, the significant risk of inclusion and attrition biases in reported cohorts cannot be overlooked, particularly given the unequal access to healthcare experienced by some refugees due to contextual factors such as political and social circumstances.

## Conclusions

This scoping review sheds light on the complex landscape of chronic pain management among refugees, emphasizing the urgent need to address systemic issues. Factors contributing to the knowledge gaps include the absence of standardized rehabilitation protocols, scarcity of interventional research on chronic pain treatment approaches and outcomes in refugees, and inadequate national legislation. However, there are avenues for progress. Research should prioritize high-quality controlled studies investigating culturally appropriate multidisciplinary pain management interventions using both quantitative and qualitative designs. Culturally sensitive care is important to bridge the gap between refugees and healthcare providers by acknowledging differing interpretations of pain and treatment preferences, and recognising the specific experiences of refugees before, during, and after forced displacement. Whilst pre-migration experiences can inform rehabilitation approaches and strategies, addressing post-migration factors provides actionable targets for change. Additionally, addressing the unique challenges faced by refugee women, whose help-seeking behaviours are often influenced by traditional gender roles and cultural norms, is essential. Clinicians can advocate for better healthcare access for refugees and publish case studies or case series to enhance understanding and share expertise. Collaboration between healthcare services and refugee organizations is essential for improving pain management outcomes. By embracing these recommendations, healthcare providers can move toward a more effective and accessible approach to chronic pain management for refugee populations, improving treatment outcomes and promoting their overall well-being.

## Supplementary Information

Supplementary Material 1.

Supplementary Material 2.

## Data Availability

All data used in this study is provided within the manuscript or additional information files.

## Abbreviations

IASP: International Association for the Study of Pain
PTSD: Post-Traumatic Stress Disorder
UNIP: United Nations Istanbul Protocol
FM: Fibromyalgia
CPP: Chronic Pelvic Pain
