# Supplementary material for: Barriers to optimal chronic pain management in refugees: a scoping review
Source: BMC Public Health. 2026 Jan 13;26:533. doi: 10.1186/s12889-026-26206-w (PMC12888163; doi:10.1186/s12889-026-26206-w)
Supplement: Supplementary file 2 — Supplementary Material 2. [file 12889_2026_26206_MOESM2_ESM.docx]

**Additional File 2**

**Search Strategy and Results for Searches conducted on 22^nd^ October 2024**

Database: **PubMed/MEDLINE**

#1 ("Refugees"[MeSH] OR "Displaced Persons"[MeSH] OR "Asylum Seekers"[MeSH] OR "Migrant*" OR "Forced Migration" OR "Humanitarian Crisis*")

#2 ("Chronic Pain"[MeSH] OR "Pain Management"[MeSH] OR "Analgesics"[MeSH] OR "Opioid Analgesics"[MeSH] OR "Non-Opioid Analgesics" OR "Persistent Pain" OR "Pain Relief" OR "Physical Therapy" OR "Rehabilitation"[MeSH] OR "Complementary Medicine")

#3 ("Intervention*" OR "Therap*" OR "Management" OR "Surgery"[MeSH] OR "Medication*")

Final: #1 AND #2 AND #3

*Results: 308*

Database: **Web of Science**

TS=("Refugee*" OR "Displaced Person*" OR "Asylum Seeker*" OR "Migrant*" OR "Forced Migration" OR "Humanitarian Crisis*")

AND TS=("Chronic Pain" OR "Persistent Pain" OR "Pain Management" OR "Pain Assessment" OR "Pain Relief" OR "Analgesia" OR "Opioids" OR "Non-Opioid Analgesics" OR "Physical Therapy" OR "Rehabilitation" OR "Complementary Medicine")

AND TS=("Treatment" OR "Management" OR "Intervention*" OR "Therap*" OR "Medication*" OR "Surgery")

*Results: 1463*

Database: **Cochrane Database of Systematic Reviews**

[Title/Abstract/Keywords]:
("Refugee*" OR "Displaced Person*" OR "Asylum Seeker*" OR "Migrant*" OR "Forced Migration" OR "Humanitarian Crisis*")

AND ("Chronic Pain" OR "Persistent Pain" OR "Pain Management" OR "Pain Assessment" OR "Pain Relief" OR "Analgesia" OR "Opioids" OR "Non-Opioid Analgesics" OR "Physical Therapy" OR "Rehabilitation" OR "Complementary Medicine")

AND ("Treatment" OR "Management" OR "Intervention*" OR "Therap*" OR "Medication*" OR "Surgery")

*Results: 54*

Database: **Scopus**

(TITLE-ABS-KEY("Refugee*" OR "Displaced Person*" OR "Asylum Seeker*" OR "Migrant*" OR "Forced Migration" OR "Humanitarian Crisis*"))

AND (TITLE-ABS-KEY("Chronic Pain" OR "Persistent Pain" OR "Pain Management" OR "Pain Assessment" OR "Pain Relief" OR "Analgesia" OR "Opioids" OR "Non-Opioid Analgesics" OR "Physical Therapy" OR "Rehabilitation" OR "Complementary Medicine"))

AND (TITLE-ABS-KEY("Treatment" OR "Management" OR "Intervention*" OR "Therap*" OR "Medication*" OR "Surgery"))

*Results: 447*

Database: **APA PsycInfo (via ProQuest)**

TI,AB,IF("Refugees" OR "Displaced Persons" OR "Asylum Seekers" OR "Migrants" OR "Forced Migration" OR "Humanitarian Crisis")

AND TI,AB,IF("Chronic Pain" OR "Persistent Pain" OR "Pain Management" OR "Pain Assessment" OR "Pain Relief" OR "Analgesia" OR "Opioids" OR "Non-Opioid Analgesics" OR "Physical Therapy" OR "Rehabilitation" OR "Complementary Medicine")

AND TI,AB,IF("Treatment" OR "Management" OR "Intervention*" OR "Therap*" OR "Medication*" OR "Surgery")

*Results: 280*
